# Designing clinical research into the treatment of breast cancer in the elderly - the advantages and challenges of a value of information approach

**DOI:** 10.1186/1745-6215-14-S1-P54

**Published:** 2013-11-29

**Authors:** Paul Richards, Alan Brennan

**Affiliations:** 1 Health Economics and Decision Science, School of Health and Related Research, The University of Sheffield, Sheffield, UK

## 

Value of information techniques form an alternative framework for study design, based on the uncertainty in long term costs and benefits associated with different treatment approaches. Given a set of candidate study designs, the optimal design is that which maximises the *expected net benefit of sampling* (*ENBS*)*.* Much contemporary discussion has focussed on the potential for using value of information in designing randomised clinical trials for drug development. However, this approach could theoretically be used to design research to improve clinical practice after treatment strategies have been adopted, where long term costs and benefits are still subject to uncertainty.

Surgery is recognised as being vital in the treatment of early breast cancer. However, in the UK many elderly women are instead treated with primary endocrine therapy. According to NICE guidance, this constitutes undertreatment. For women with limited life expectancy or frailty, less intensive therapeutic approaches may be beneficial. However, more research is required to ensure that treatments are chosen appropriately.

This poster presents a case study of using value of information methods to design a study to address this problem. The use of Bayesian evidence synthesis techniques and simulation allow for the calculation of ENBS of a wide range of study designs, including experimental and observational designs. A key methodological challenge in this case study is specifying a sensible representation of current beliefs about heterogeneity in outcomes given limited evidence. The computational cost of these methods remains a barrier to implementation, though more efficient algorithms are in development.

